# “Bicycles May Use Full Lane” Signage Communicates U.S. Roadway Rules and Increases Perception of Safety

**DOI:** 10.1371/journal.pone.0136973

**Published:** 2015-08-28

**Authors:** George Hess, M. Nils Peterson

**Affiliations:** Department of Forestry & Environmental Resources, NC State University, Raleigh, North Carolina, United States of America; University of New South Wales, AUSTRALIA

## Abstract

Many global challenges, including obesity, health care costs, and climate change, could be addressed in part by increasing the use of bicycles for transportation. Concern about the safety of bicycling on roadways is frequently cited as a deterrent to increasing bicycle use in the USA. The use of effective signage along roadways might help alleviate these concerns by increasing knowledge about the rights and duties of bicyclists and motorists, ideally reducing crashes. We administered a web-based survey, using Twitter for recruitment, to examine how well three US traffic control devices communicated the message that bicyclists are permitted in the center of the travel lane and do not have to “get out of the way” to allow motorists to pass without changing lanes: “Bicycles May Use Full Lane” and “Share the Road” signage, and Shared Lane Markings on the pavement. Each was compared to an unsigned roadway. We also asked respondents whether it was safe for a bicyclist to occupy the center of the travel lane. “Bicycles May Use Full Lane” signage was the most consistently comprehended device for communicating the message that bicyclists may occupy the travel lane and also increased perceptions of safety. “Share the Road” signage did not increase comprehension or perceptions of safety. Shared Lane Markings fell somewhere between. “Bicycles May Use Full Lane” signage showed notable increases in comprehension among novice bicyclists and private motor vehicle commuters, critical target audiences for efforts to promote bicycling in the USA. Although limited in scope, our survey results are indicative and suggest that Departments of Transportation consider replacing “Share the Road” with “Bicycles May Use Full Lane” signage, possibly combined with Shared Lane Markings, if the intent is to increase awareness of roadway rights and responsibilities. Further evaluation through virtual reality simulations and on-road experiments is merited.

## Introduction

Many of the greatest challenges facing humanity globally can be addressed, in part, by bicycling.

This statement may seem like exaggeration, but under scrutiny may be an understatement [1]. For instance, obesity is tied to car dependency in the United States and linked to the first lifespan decline in 200 years for people living there [2–3]. Cyclists are healthier and spend less time and money on medical care than other commuters [4]. Bicycling can promote mobility, particularly for the poor and elderly who often live in landscapes with amenities too dispersed for pedestrian access and cannot afford personal motor vehicle travel [5]. Replacing a car with a bicycle is, by a large margin, the single most important change a person can make to reduce their contributions to climate change [5–6]. Finally, because average Americans spend 15.6% of their income on motor vehicle driving, riding bicycles could have a large, positive effect on economic welfare and overall quality of life [5,7].

Why hasn’t this incredibly simple solution to so many problems been more broadly adopted, particularly in the United States? The reasons are complex and intertwined, including lack of access or desire to bicycle, roadways designed primarily for motor vehicle traffic, unpleasant weather, route, and light conditions, the need to carry bulky or heavy loads, and safety concerns [1,8–9]. In this article, we focus on safety concerns.

Most US cities have horrible bicycle safety records, with mortality rates about double those in other developed nations and injury rates between eight and 30 times higher [10]. Perceptions of unsafe conditions for bicycling are cited as an important deterrent to initiating or increasing bicycle use in many studies [8–9,11–12]. In a survey of Texas bicyclists, for example, Sener and others found that 69% felt bicycling was somewhat or very dangerous from the perspective of potential traffic crashes [13]. In particular, motorists driving too close (40%) or too fast (32%) were the most frequently reported dangers among the 11% of bicyclists who felt threatened by motorists [8,12].

Research consistently demonstrates that some infrastructure promotes safety, including traffic calming features, using separate bicycle paths, or placing barriers between motor vehicle and bicycle traffic [14]. Bicyclists in the US will invariably have to share roadway travel lanes with motor vehicles, however, because most roadways do not have bicycle-specific infrastructure. In fact, Royal and Muller-Steiger reported that 61% of US bicycle trips were made on paved roads (including their shoulders) without such infrastructure [8]. The lanes on most US roadways are too narrow for motorists to pass bicyclists within the same lane.

**Fig 1:**
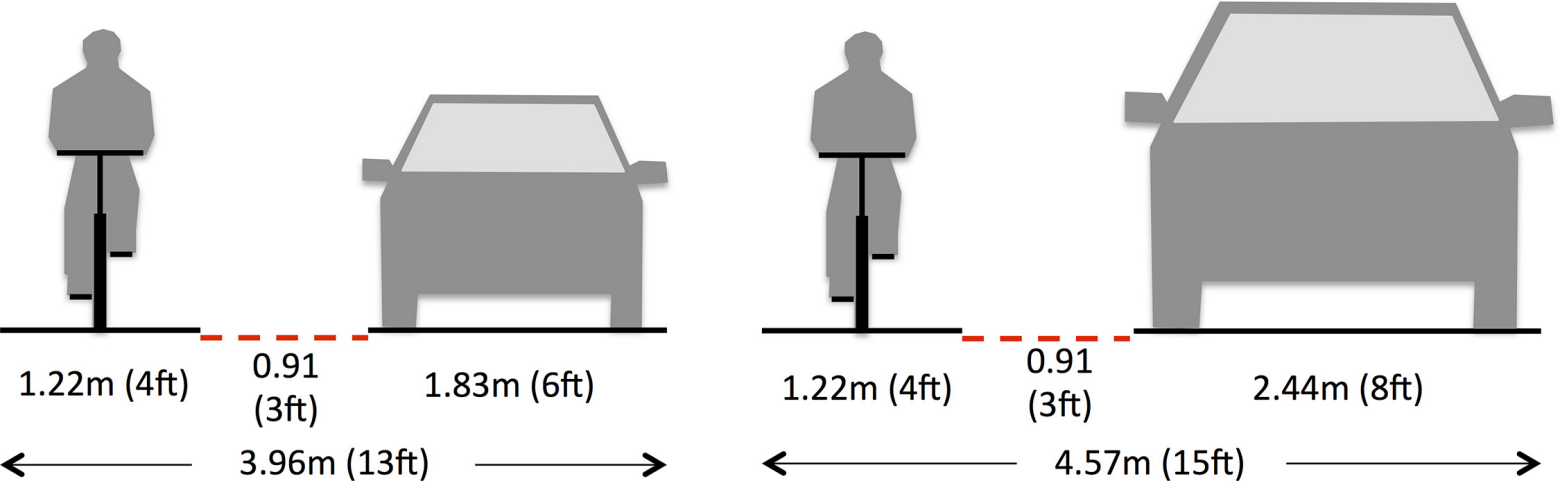
Geometry of lawfully passing a bicyclist. Depiction of two common US motor vehicles (typical sedan on the left, pickup truck/sport utility vehicle on the right), 0.91m (3ft) minimum lateral passing distance required in most states (red dashed line), and typical 1.22m (4ft) operating space for bicyclists. The resultant total width does not include any “shy distance,” typically 0.46m (1.5ft) that motorists and bicyclists keep away from hazardous objects, between the motor vehicle and the lane marking to its left, or between the bicyclist and the edge of the road.

A 4.27m (14ft) travel lane is generally recognized as the narrowest in which motorists can pass bicyclists within the same lane, with 4.57–4.88m (15–16ft) needed where speeds, overall traffic volume, or large truck traffic volume are higher (Fig 1) [15–17]. US roadways typically have narrower travel lanes, stripped between 3.05–3.66m (10–12ft) in width, requiring motorists to move fully or partially into the adjacent lane to lawfully pass a bicyclist. Numerous bicycle safety education programs teach bicyclists that riding near the edge of typical roadways encourages unlawful, too-close passing and that they should ride near the center of the travel lane to encourage motorists to change lanes before passing [18–19]. This engenders resentment among motorists who must wait behind slow-moving bicycle traffic, and close passes, harassment, and fear of overtaking collisions among bicyclists, particularly those with inadequate skills and knowledge of bicycling in traffic; bicyclists have also reported a pattern of harassment by motorists and some police when practicing this technique [20].

Traffic control devices might improve interactions among bicyclists and motorists on US roadways, improve the perception and reality of bicyclist safety, and ultimately lead to increased bicycling. Ideally, such traffic control devices would increase roadway users’ knowledge of the rights of bicyclists on roadways, help motorists recognize the need to change lanes when passing bicyclists, support the practice of bicyclists using the full travel lane under conditions where motorists cannot pass bicyclists within the lane, and reduce the number of crashes. Put plainly, they would communicate the message that bicyclists are permitted in the travel lane and do not have to “get out of the way” to allow motorists to pass without changing lanes. We examined the potential of three traffic control devices to attain these goals on US roadways: “Share the Road,” “Bicycles May Use Full Lane,” and Shared Lane Markings [21].

### Traffic Control Devices for Bicycles as Vehicles

Bicycles are classified or treated as vehicles in all 50 US states, which means that bicyclists have most of the same legal operating rights and responsibilities as motorists, including the right to occupy a full travel lane [22]. North Carolina’s Driver Manual states this unambiguously: “Bicyclists usually ride on the right side of the lane, but are entitled to use of a full lane” [23]. In many states, so-called “far-to-right” laws specifically require bicyclists to drive as far to the right as practicable. Yet, even in those states bicyclists ultimately have the right to use the full lane when, at their discretion, driving far-to-right is not practicable. Florida’s Driver Manual, for example, notes that “A bicyclist may use the full lane even while traveling substantially below the speed of traffic if the lane is too narrow for a car to safely pass a bicycle within the lane” [24].

**Fig 2:**
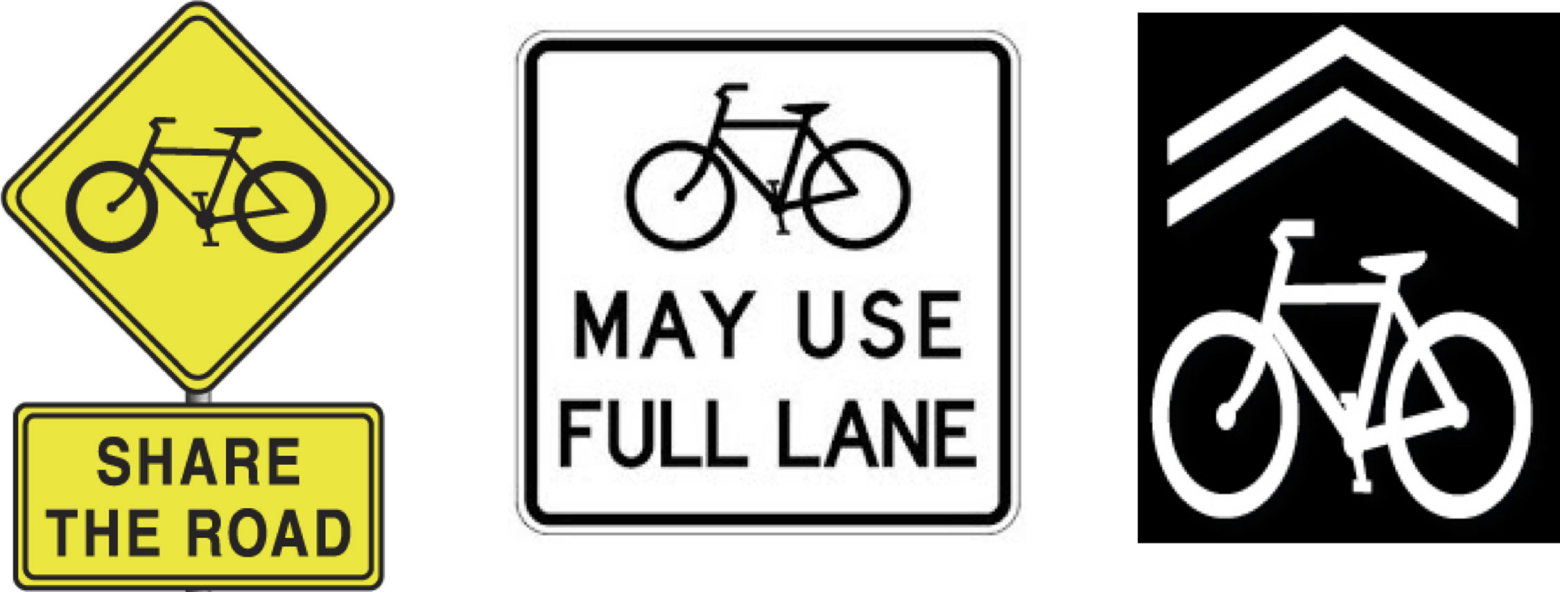
Bicycle-related traffic control devices. From left to right, “Share the Road” (Manual on Uniform Traffic Control Devices W11-1 upper plaque plus W16-1 lower) and “Bicycles May Use Full Lane” (R4-11) signage; Shared Lane Markings (sharrows) are painted on the roadway pavement [21].

The right of bicyclists to use a full travel lane is not well-recognized by motorists or bicyclists, which contributes to safety concerns and creates social friction among them [25]. State Departments of Transportation have used three traffic control devices in attempts to communicate the right of bicyclists to use roadways, most commonly “Share the Road” signage and, more recently, “Bicycles May Use Full Lane” signage and Shared Lane Markings, also called “sharrows,” on the roadway pavement (Fig 2) [21]. The two-plaque “Share the Road” signage was designed to warn motorists that slow-moving vehicles–in this case bicycles–may be on the roadway [21]. Many State Departments of Transportation also declare that it is intended to remind motorists that bicyclists have a legal right to use roadways, though it was not designed for that purpose [26]. “Bicycles May Use Full Lane” signage is intended to remind roadway users that bicyclists might be in the travel lane and have a legal right to occupy the full lane. Shared Lane Markings on roadway pavement are intended to suggest appropriate lane positioning for bicyclists, alert other roadway users that bicyclists may be present within the travel lane, and encourage safe passing of bicyclists by motorists.

Basic design principles provide guidance for hypotheses regarding traffic control devices, which must be clearly legible and quickly comprehended [21,27]. Our focus is on comprehension, which for signage in general emerges from “guessability,” learnability, and experienced user performance [28]. Comprehensible traffic signs are concrete (visually obvious, not abstract) and simple (few elements and little detail), which make them meaningful; they also have low semantic distance (closeness between what is depicted and what it is intended to represent; for example, a picture of a bicycle to suggest bicycles ahead has a lower semantic distance than the use of a triangle to suggest a hazard) and are familiar (frequently encountered) [29]. Effective signs should be comprehensible, unambiguous, precise [30], and “convey a clear, simple meaning” [21].

Comprehension of the familiar “Share the Road” signage as a statement of bicyclists’ roadway rights has been challenged, based on arguments that it is ambiguous, imprecise, frequently misinterpreted, and not designed for that purpose [31–32]. Although often described as a reminder to motorists that bicyclists may use the travel lane [26], bicyclists frequently complain that motorists interpret the sign to mean that they should get out of the way. In fact, the US state of Delaware discontinued use of the “Share the Road” plaque in November, 2013, because “Some believe the plaque puts more onus on the bicyclist to share the road than the motorist” [33]. Those challenging “Share the Road” signage often call for the use of “Bicycles May Use Full Lane” instead, because it is an unambiguous, precise statement of law [32]. Shared Lane Markings are intended to show bicyclists the best place to ride and remind motorists that bicyclists are permitted in the travel lane [21], but may be somewhat ambiguous and imprecise in communicating that message.

Evaluations of the comprehensibility of “Share the Road” and “Bicycles May Use Full Lane” signage in the USA are few and have produced conflicting results, in part because roadway conditions introduce confounding factors (**S1 Text**). Studies of Shared Lane Markings are more common, also have conflicting results, and have been subject to criticism for drawing positive conclusions from the evaluation of improperly placed markings or using inappropriate metrics of success.

### Objective

Our contribution to the literature is a straightforward evaluation of the comprehensibility of three traffic control devices: “Share the Road” and “Bicycles May Use Full Lane” signage and Shared Lane Markings. We evaluated whether any of these devices increased awareness among roadway users, relative to a control situation with no traffic control devices, that bicyclists are permitted to occupy the full travel lane and that motorists should wait to pass only after moving into the adjacent lane. Secondarily, we evaluated perceptions of safety in response to each traffic control device and with no device.

We chose to evaluate these traffic control devices based on design principles and pragmatic grounds. Pragmatically, these are the four most common situations faced by US cyclists. From the perspective of design principles, although “Share the Road” signage is the most familiar, we hypothesized it would not increase comprehension, relative to an unsigned roadway, of bicyclists’ right to use the travel lane because of its high semantic distance and ambiguous, imprecise message. We hypothesized that “Bicycles May Use Full Lane” signage would increase comprehension because it is meaningful, unambiguous, precise, and has low semantic distance; and that Shared Lane Markings would somewhat increase comprehension because the markings are concrete, but ambiguous and imprecise with medium semantic distance. Our hypotheses and expectations were rooted in previous research suggesting semantic distance was more important than familiarity in terms of eliciting correct interpretation of signs and symbols [34–35].

## Methods

We used a web-based survey to examine the comprehensibility of three traffic control devices on roadways relative to unsigned roadways using unambiguous scenarios and simple questions. Comprehensibility was measured by the degree to which the device increased, when compared to no device, respondent recognition of the right of bicyclists to occupy the full travel lane and the duty of motorists to move into the adjacent lane to pass.

### Survey Recruitment

**Fig 3:**
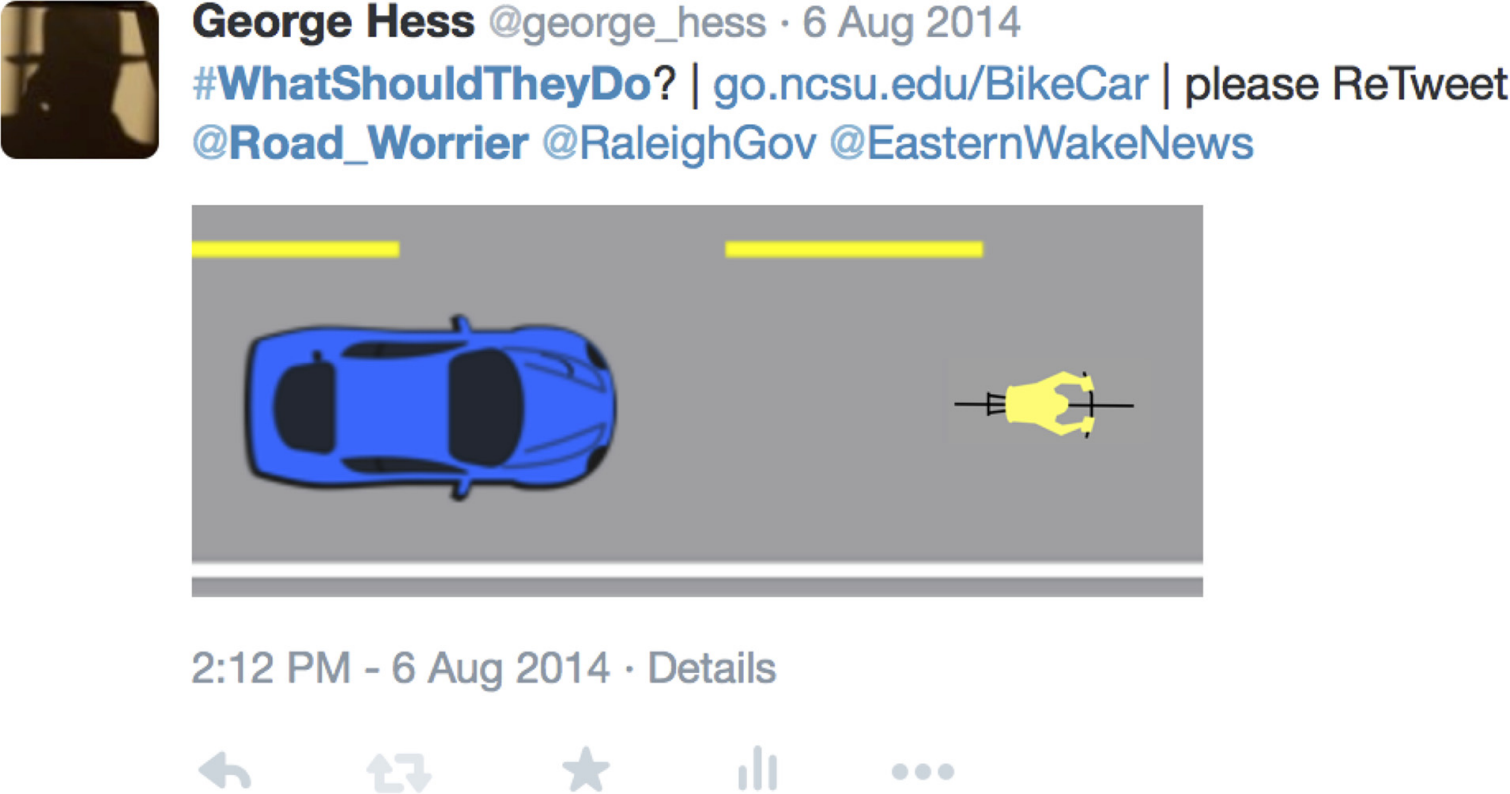
Example tweet. Each tweet included a request that the viewer complete our survey and retweet it and was directed at other Twitter users (the @handles) and Twitter users interested in specific issues (keywords preceded by #).

We conducted a web-based survey using a convenience sample of respondents recruited through a series of tweets–short messages sent through Twitter (twitter.com), a social media tool (Fig 3). Tweets were originated in waves by Twitter user @george_hess and directed to (“mentioned,” in the language of Twitter) other Twitter users with an interest in automobiles, bicycles, highway safety, and mass transportation, and using several different hashtags (keywords preceded by #) representing the same interests. Recipients were asked to retweet widely, to further spread our request for survey respondents. In a few cases, Hess contacted Twitter users by electronic mail to explain the survey and ask them to retweet. Our questionnaire was designed to be completed on computers, tablets, and mobile phones in well under 5 minutes.

Before administering the survey, we conducted two pilot tests. The first involved cognitive interviews [36] with four people knowledgeable about driving bicycles on roadways and aware of the full study design. During the second, we engaged a group of six naïve respondents who pre-tested the survey in the manner it would be seen during the study. We modified the survey after each pilot test to increase clarity, streamline flow, and ensure statistical rigor. The survey was opened on 14 July 2014 and closed on 19 September 2014.

### Ethics Statement

Our survey protocol was reviewed and approved by the NC State University Institutional Review Board for the Protection of Human Subject in Research (Study #4070) during June, 2014.

### Questionnaire Design

**Fig 4:**
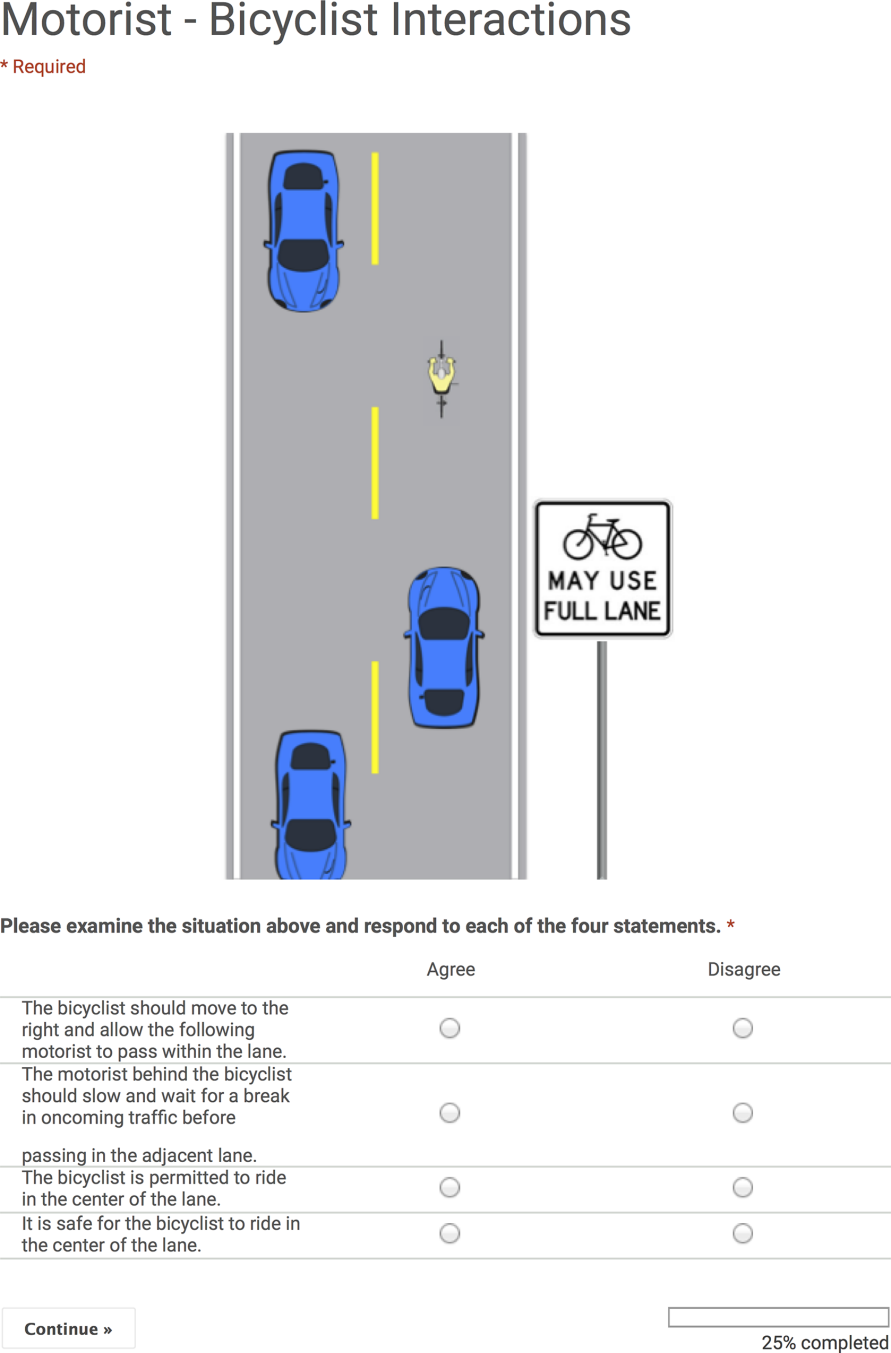
Example traffic situation. This shows the image and statements we used for the two-lane roadway with the *Bicycles May Use Full Lane* signage treatment. Images for the other three treatments were identical except for signage, which was appropriate for the treatment. The full survey is shown in **S1 Methods**; images for all four treatments are reproduced in **S2 Methods**.

In our questionnaire, respondents were asked to evaluate two traffic situations involving motor vehicles and a bicycle: a two-lane and a four-lane roadway. The situations each depicted a motorist behind a bicyclist riding in the center of the travel lane (Fig 4). The roadway was depicted intentionally with lanes too narrow for lawful in-lane passing of bicyclists by motor vehicles (approximately 3.35m (11ft) travel lane, 1.83m (6ft) wide motor vehicle), no shoulder, no adjacent bicycle or mutli-use path, passing permitted (dashed lane lines), and traffic in the adjacent lane precluding immediate, lawful passing of the bicyclist by the motorist. This depiction was intended to focus attention on essential questions of respondents’ beliefs about the rights and duties of the bicyclist and following motorist, without confounding factors such as the possibility of the bicyclist using a shoulder, the presence of a no-passing zone, and the absence of traffic in the adjacent travel lane.

For each of the two traffic situations, respondents were asked to agree or disagree with four statements regarding action the motorist or bicyclist should take, and the permissibility and safety of the bicyclist’s road position. We created four versions of the questionnaire, one for each of our three signage scenarios and a control with no signage. All versions of the questionnaire were identical with the exception of the images for the two traffic situations; the images for each treatment contained the appropriate signage or travel lane markings (Fig 4, **S1 and S2 Methods**). The first panel of the questionnaire was an informed consent and an invitation for eligible respondents to proceed to the survey. To avoid drawing attention to the signage, the informed consent indicated that respondents were being asked to evaluate motorist–bicyclist interactions. Once a potential respondent proceeded, s/he was served, at random, one of the four versions of the questionnaire (**S2 Methods**).

We also asked respondents to provide information about the distance they drove motor vehicles and bicycles weekly, primary mode of transportation to school or work, level of education, gender, and state of residence. Responses to all demographic questions except education and gender were required for questionnaire submission.

### Data Analysis

We obtained 1,978 responses to our questionnaire (Table 1) and excluded 154 from our analysis, because the respondents identified themselves as living outside the United States. We analyzed the remaining 1,824 responses (**S1 and S2 Data**) using a logistic regression model that predicted agreement versus disagreement with each statement as a function of the signage scenario (each of the three traffic control devices compared to no signage), the respondent’s primary commute mode (private motor vehicle vs. other), and the distance the respondent bicycled during a typical week (≤16km(10mi) vs. >16km). Our analyses were conducted using the statistical package, R (www.r-project.org).

**Table 1:** Characteristics of our survey respondents.

**Geographic Distribution**		**Traffic Control Device**	
United States	1,824 (92%)	Bicycle May Use Full Lane	489 (27%)
Non-US (excluded from analyses)	154 (8%)	Shared Lane Marking	454 (25%)
Total	1,978 (100%)	Share the Road	422 (23%)
		None	459 (25%)
		Total	1,824 (100%)
**Weekly Cycling Distance**		**Commute Mode**	
≤16km (10mi)	369 (20%)	Private Motor Vehicle	776 (43%)
>16km	1,455 (80%)	Other means	1,048 (57%)
Total	1,824 (100%)	Total	1,824 (100%)
**Education**		**Gender**	
Less than High School	3 (<1%)	Female	411 (23%)
High School	133 (7%)	Male	1,387 (76%)
Community College	171 (9%)	No response	26 (1%)
4-Year College	843 (46%)	Total	1,824 (100%)
Graduate Degree	667 (37%)		
No response	7 (<1%)		
Total	1,824 (100%)		

## Results

**Fig 5:**
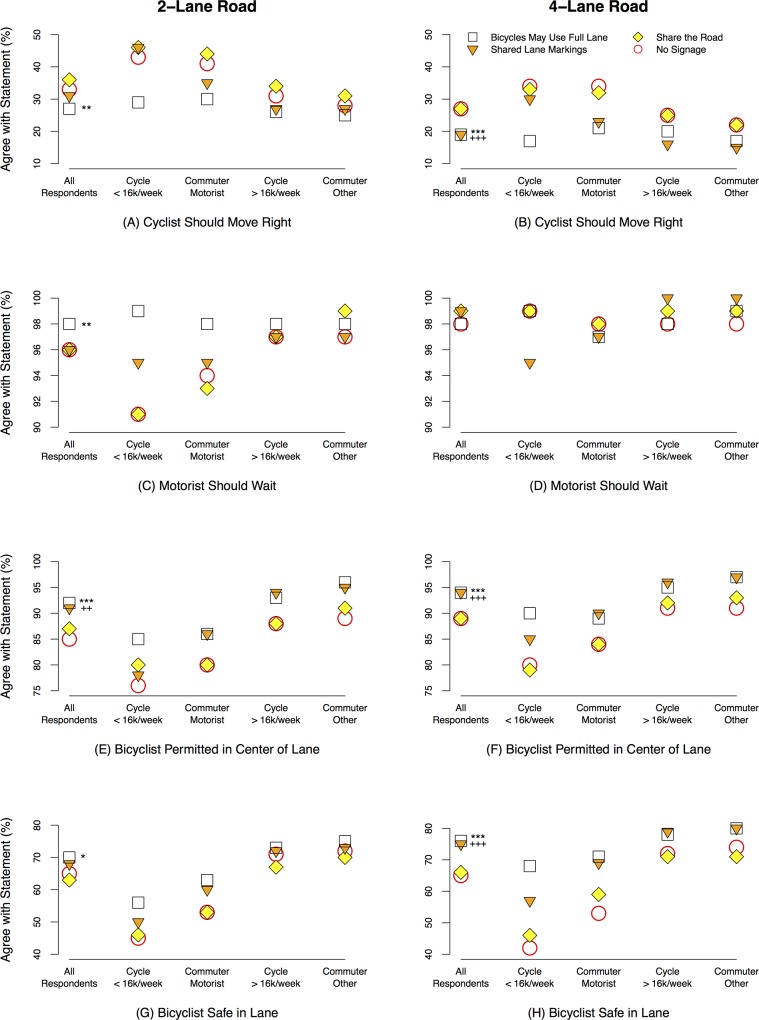
Comparison of agreement with four road use statements under three treatments. Bicycle May Use Full Lane–square; Shared Lane Markings–inverted triangle; Share the Road–yellow diamond; and control (No Sign)–red circle on 2-lane (left column) and 4-lane (right column) roads. In each plot results for the overall sample (n = 1,824) are compared to respondents who bicycle ≤ 16km/week (n = 369), who commute by personal motor vehicle (n = 776), who bicycle > 16km/week (n = 1,455), and who commute by other means (n = 1,048). The y-axis scales vary among statements (rows). For all respondents, significant differences between the Bicycles May Use Full Lane treatment and the control (No sign) are marked using * for p<0.1, ** p<0.05, *** p<0.01; similarly, for the Shared Lane Marking treatment they are marked using +; Share the Road never differed significantly from no signage.

In almost every case, on 2- and 4-lane roadways, respondents who saw “Bicycle May Use Full Lane” signage were significantly more likely (p<0.1) than those who saw no signage to agree that bicyclists are permitted in the center of the lane, do not have to move right to allow motorists to pass within the same lane, that motorists should wait for a break in traffic before passing in the adjacent lane, and that bicyclists are safe in the travel lane (Fig 5, Table 2, **S1 Table**). The only exception was on 4-lane roadways in response to the statement that motorists should wait to pass, for which behavior was unaffected by the signage. Agreement with this statement, however, was uniformly high (≥95%), regardless of traffic control devices, suggesting the vast majority of roadway users recognize the duty of motorists to wait for an opportunity to pass even without signage.

**Table 2:** Comparison of agreement with four road use statements by treatment and by two user categories on 2- and 4-lane roads.

	*Treatments*			*User Categories*	
Statement	Share the Road	Shared Lane Markings	Bicycle May Use Full Lane	Cycle >16k/week	Commute by Other Than Motor Vehicle
***2-lane Road***					
The bicyclist should move to the right and allow to following motorist to pass within the lane.	1.16	0.89	**0.73 − −**	**0.69 − − −**	**0.70 − − −**
The motorist behind the bicyclist should slow and wait for a break in oncoming traffic before passing in the adjacent lane.	1.12	1.13	**2.25 ++**	**1.77 ++**	**1.82 ++**
The bicyclist is permitted to ride in the center of the lane.	1.11	**1.69 ++**	**1.96 +++**	**1.92 +++**	**2.20 +++**
It is safe for the bicyclist to ride in the center of the lane.	0.90	1.12	**1.28 +**	**2.11 +++**	**1.65 +++**
***4-lane Road***					
The bicyclist should move to the right and allow to following motorist to pass within the lane.	0.99	**0.63 − − −**	**0.62− − −**	**0.78 −**	**0.66 − − −**
The motorist behind the bicyclist should slow and wait for a break in traffic before passing in the adjacent lane.	1.46	1.91	1.20	1.12	**2.20 +**
The bicyclist is permitted to ride in the center of the lane.	1.01	**1.99 +++**	**1.98 +++**	**2.09 +++**	**2.20 +++**
It is safe for the bicyclist to ride in the center of the lane.	1.00	**1.60 +++**	**1.68 +++**	**2.33 +++**	**1.53 +++**

For treatments (Share the Road, Shared Lane Markings, Bicycle May Use Full Lane) the comparison is to the control case of no signage; the odds ratio shown compares each treatment to the control. For user categories, the comparison and associated odds ratio, is to the contrasting user category (i.e., respondents who cycle >16k/week vs. those who cycle≤16k/week; those who commute by means other than personal motor vehicle vs. those who commute by personal motor vehicle). All significant differences are shown in bold font and followed by signs that show whether tendency to agree increased (+) or decreased (-) relative to the comparison case. The number of signs indicates the significance level: 1 sign p<0.1; 2 signs p<0.05; 3 signs p<0.01. Cells in normal font and without following signs indicate no significant difference (p≥0.1) from the comparison case. Full model results (parameter estimates, standard error, p-values) are presented in **S1 Table**.

There was no statistically significant difference in responses between those who saw “Share the Road” signage and those who saw no signage in any scenario we tested (Fig 5, Table 2).

Respondents who experienced Shared Lane Markings were significantly more likely than respondents who saw no signage to agree that bicyclists are permitted in the center of the travel lane, on both 2- and 4-lane roads (Table 2). They were also more likely, on 4-lane roads, to disagree that the bicyclist had to move out of the way and to agree that the bicyclist was safe in the center of the lane.

Regardless of signage, respondents who commuted to work by means other than personal motor vehicle were significantly more likely than those who commuted by personal motor vehicle to agree that bicyclists are permitted in the center of the lane, do *not* have to move right to allow motorists to pass within the same lane, that motorists should wait for a break in traffic before passing in the adjacent lane, and that bicyclists are safe in the travel lane (Table 2). The same can be said of respondents who bicycled >16km/week in comparison to those who bicycled ≤16km/week, except on 4-lane roads for which there was no difference between the groups in response to the “motorist should wait to pass” statement.

We saw the largest shifts in response to the “Bicycles May Use Full Lane” signage when compared to no traffic control devices among respondents who bicycled ≤16km per week or commuted by private motor vehicle (Fig 5). Among those who bicycled ≤16km per week, for example, agreement with the statement that the bicyclist should move right to allow the motorist to pass within the same lane decreased 14 percentage points (to 29%) on the 2-lane roadway and 17 percentage points (to 17%) on the 4-lane roadway, compared to 5-point decreases (to 26% and 20%) for respondents who bicycled >16km per week. Among respondents who commuted by private motor vehicle we saw a 10-point increase (to 63%) in agreement with the statement that it was safe for the bicyclist to be in the center of the travel lane on 2-lane roadways, and an 18-point increase (to 71%) on the 4-lane roadway, compared to 3 points (to 75%) and 6 points (to 80%) for respondents who commuted by other means.

Although significantly more respondents agreed that bicyclists are permitted in the center of the travel lane when presented “Bicycles May Use Full Lane” signage, a large proportion still disagreed – 8% overall on 2-lane and 6% on 4-lane roadways. The tendency to disagree was particularly strong among those who cycled ≤16km per week (15% on 2-lane and 10% on 4-lane roadways) or commuted by private motor vehicle (14% on 2-lane and 11% on 4-lane roadways).

## Discussion

Taken as a whole, our results supported our hypotheses. “Bicycles May Use Full Lane” signage was the most comprehensible traffic control device of those we tested for delivering the message that bicyclists may use a full lane and do not have to “get out of the way” for motorists, and for increasing the perception that using the full lane is safe. “Share the Road” signage provided no additional comprehension compared to an unsigned roadway; this is unsurprising because, despite being used for that purpose [26], it was not designed to do so [21]. Shared Lane Markings on roadway pavement fell somewhere between, showing statistically significant effects in some cases but not others.

Our results are consistent with sign design principles and guidelines promulgated in the Manual on Uniform Traffic Control Devices that support concrete, simple, unambiguous, and precise signage with low semantic distance that “convey a clear, simple meaning” [21,29–30]. They support arguments that “Share the Road” signage should not be used as a substitute for “Bicycles May Use Full Lane” signage to deliver that message, because “Share the Road” is imprecise and ambiguous for that purpose. While the one available study we reviewed for “Bicycles May Use Full Lane” signage (**S1 Text**) showed mixed results, we believe they can be ascribed to the confounding factors described in that study rather than inherent problems with comprehensibility of the signage. Our middle-of-the-road results for Shared Lane Markings suggest comprehensibility issues that may be partly responsible for conflicting results in on-road observational studies, many of which also suffered from improper placement of the Shared Lane Markings (**S1 Text**). The Manual on Uniform Traffic Control Devices suggests that “Bicycles May Use Full Lane” signage and Shared Lane Markings may be used together [21]. Although we did not test this combination, our results lead us to hypothesize that the combination would be more comprehensible than either device used in isolation.

The improved perceptions of safety elicited by “Bicycles May Use Full Lane” signage suggest that its expanded use could help increase cycling rates. Specifically, because concerns about safety are a deterrent to initiating or increasing bicycling [9,11–12], signage improving perceptions of safety may increase bicycling mode share. “Bicycles May Use Full Lane” signage significantly increased perceptions of safety when bicycling in the travel lane on 2- and 4-lane roadways, whereas Shared Lane Markings did so only on 4-lane roadways; “Share the Road” signage did not differ significantly in this regard from the roadways without traffic control devices.

The comparative advantages of “Bicycle May Use Full Lane” signage, and failure of “Share the Road” signage, may be much larger than our results suggest, because our sample included an unusually high number of bicycle commuters and bicycle riders relative to the US population (Table 1). Further, the positive effects, in terms of delivering the message and increasing perceptions of safety, of “Bicycle May Use Full Lane” signage were particularly strong for the two groups of respondents who most accurately reflect the US population: the 86% who commuted by private motor vehicle in 2013 [37] and those who do not bicycle >16km per week. These portions of the population are likely to be less familiar with the application of vehicle codes to bicyclists and represent critical target audiences for traffic control devices and efforts to promote bicycling in the US.

Our results show a clear, consistent pattern of greater importance of these bicycle-related traffic control devices among private motor vehicle commuters and respondents with less experience bicycling (Fig 5). We speculate that as people learn through real-life experience–by bicycling more or by commuting in ways other than private motor vehicle–they no longer need signs to remind them of appropriate behavior. One way of learning is through clear messages delivered by well-designed signage, which, in our study, appear to have influenced the views of novice bicyclists and private motor vehicle commuters. An effective bicycle-related traffic control device could initiate a virtuous cycle, because small increases in the number of bicycle commuters can create large declines in risk as bicyclists become part of motorists’ search image. In California, for example, the risk of a bicyclist being hit by a motor vehicle is about ten times lower in communities where more than 2% of the residents commute on bicycles, relative to places where less than 1% do [38]. Decreasing risk could lead to even further increasing bicycle use.

The reasons for low bicycle use in the US are multiple and complex, including land use patterns that spread destinations too far apart for bicycle trips of reasonable length; roadways designed primarily for motor vehicle traffic; failure, or even refusal, of motorists to recognize the right of bicyclists to use a full travel lane; and failure of bicyclists to follow traffic regulations. These factors contribute to safety concerns among all roadway users and create social friction among motorists and bicyclists. While we do not expect signage alone to turn the US into a bicycling haven, well-designed signage that conveys the intended message in a clear and simple way could be an important and highly visible part of a broader educational campaign on roadway users’ rights and responsibilities, potentially improving conditions for bicyclists.

Our findings suggest that Departments of Transportation should evaluate replacing “Share the Road” signs–which are already located in areas of potential motorist-bicyclist conflict–with “Bicycles May Use Full Lane” signs to provide a less ambiguous, more educational statement, with no net increase in visual clutter. There is potential for backlash, however, from motorists frustrated by learning they are legally obligated to wait behind bicyclists; this could lead to increased harassment of bicyclists and banning bicycles and other slow-moving vehicles from roadways. For example, North Carolina’s Commissioner of Motor Vehicles recently recommended banning mopeds from roadways with speed limits of 45 miles per hour or faster [39]; it is not hard to imagine a similar recommendation for bicycles.

### Study Limitations

We evaluated comprehensibility only, and in a limited and highly controlled situation. In real-world traffic situations, reaction time, visibility, and visual clutter may make sign interpretations more difficult for roadway users. This is especially true in urban areas, where such devices abound and may only be visible briefly [27]. While recognizing these limitations, we argue that a traffic control device with low comprehensibility in a highly controlled situation such as our survey is unlikely to be effective on real roadways where these additional factors must be considered.

Using Twitter to recruit survey respondents limits our scope of inference, because Twitter users are not representative of the US population. For example, Mislove and others reported that, in the US, Twitter users overrepresented densely populated areas and were not a representative sample of gender or ethnicity [40]. Our respondent population was skewed toward people who cycled >16km per week (80%) and commuted by other than private motor vehicle (57%) (Table 1). Only 165 (9%) respondents reported that they did not bicycle at all during a typical week (these are included with our ≤16km per week respondents). This happened for at least two reasons. First, bicyclists seemed to have more of a vested interest in the outcome and consequently spread the survey link widely to friends and to groups to which they belonged. Second, and related, a popular urban bicycling blog, UrbanVelo (urbanvelo.org), posted an entry about our survey which attracted bicyclists. Our findings, however, would likely be stronger with a less biased sample because the different impacts of signs found in this study were strongest among non-cyclists.

### Further Work

Our finding that signage had the greatest effect on understanding of traffic laws and perceptions of safety among respondents who cycled the least suggests that future research should target people who do not cycle at all, as these roadway users may benefit most from more comprehensible signage. Limiting the scope of future studies to assessing “Bicycles May Use Full Lane” signage and Shared Lane Markings would further decrease the sample size needed to evaluate differences related to key demographic attributes not considered in this study, such as child versus adult. Although “Share the Road” signage was no better than an unsigned roadway at conveying the message that bicyclists are permitted to occupy a full lane, future studies may find it conveys other important messages.

The use of emerging virtual reality technologies that allow respondents to experience realistic traffic situations using immersive, head-mounted displays can further enhance our ability to evaluate traffic control devices [41]. Respondents would be put behind the wheel of a virtual motor vehicle or handlebars of a virtual bicycle and exposed to a variety of roadway conditions. This would add reaction time, visibility, and visual clutter in a controlled environment with replication–every respondent would experience the exact same situations, reducing the complexity and confounding factors of on-road observation. Eye tracking would allow researchers to better understand what respondents are observing, and it would also be possible to collect supplemental data about knowledge and experience from each respondent.

On-road experiments, while an important part of evaluating traffic control devices, are inherently difficult to conduct, replicate, and compare to a control treatment. Even comparing different signage on different roadways is perilous, because each roadway has its own peculiarities, uses, and users that may confound comparisons; and it is difficult to collect data from the bicyclists and motorists using the roads. As in previous evaluations, fixed cameras could be used to measure interactions between bicyclists and motorists. We can also envision equipping experienced bicyclists with cameras and electronic distance measuring devices, and instructing them to ride up and down test roadways in varied, measurable, and repeatable ways to measure motorist behavior.

## Conclusion

Of the three bicycle-related traffic control devices we tested, “Bicycles May Use Full Lane” signage delivered the message about the rights and responsibilities of bicyclists and motorists with respect to travel lane occupancy most consistently: bicyclists are permitted in the travel lane and need not move to allow motorists to pass them within the lane. Although Shared Lane Markings did increase comprehension in some cases, they did not deliver the message as consistently as “Bicycles May Use Full Lane” signage. We speculate that a combination of “Bicycles May Use Full Lane” signage and Shared Lane Markings might be particularly comprehensible. “Share the Road” signage failed to provide any additional comprehension in this regard when compared to the unsigned roadways in any of our tests. “Bicycles May Use Full Lane” showed particularly strong increases in comprehension for novice bicyclists and private motor vehicle commuters, critical target audiences for these traffic control devices and for efforts to promote bicycling in the US.

## Supporting Information

S1 Data Data dictionary. List and description of variables in dataset (see **S2 Data** for full dataset).
(PDF)

S2 Data Comma-separated variable (CSV) dataset used in our analyses. (DAT)

S1 Methods Complete survey, shown here for the Bicycles May Use Full Lane treatment. Each screen from the survey is shown separately as the respondent would experience them.
(PDF)

S2 Methods Images for all four treatments, shown here for the two-lane roadway. (PDF)

S1 Table Parameter estimates, standard errors, and p-values for our logistic model. (PDF)

S1 Text Review of studies of the three traffic control devices evaluated in this article. (PDF)
