# Supplementary material for: “Bicycles May Use Full Lane” Signage Communicates U.S. Roadway Rules and Increases Perception of Safety
Source: PLoS One. 2015 Aug 28;10(8):e0136973. doi: 10.1371/journal.pone.0136973 (PMC4552809; doi:10.1371/journal.pone.0136973)
Supplement: S1 Data — List and description of variables in dataset (see S2 Data for full dataset). (PDF) [file pone.0136973.s001.pdf]

S1 Data. Data Dictionary. List and description of variables in dataset (see **S2 Data**).

Each variable in our comma-separated variable (CSV) dataset is described below in a consistent format.

Variable Name: Question or description.
Possible responses, separated by semicolons (;).

Treatment: The traffic control device the respondent viewed.
1_None (no signage); 2_STR (Share the Road signage); 3_SLM (Shared Lane Markings); 4_BMUFL (Bicycles May Use Full Lane signage)

Timestamp: Date and time the survey was submitted.
MM.DD.YYY HH:MM:SEC (24-hour time)

BikeMoveRight2: [2-lane roadway] The bicyclist should move to the right and allow the following motorist to pass within the lane.
0_Disagree; 1_Agree

MVWait2: [2-lane roadway] The motorist behind the bicyclist should slow and wait for a break in incoming traffic before passing in the adjacent lane.
0_Disagree; 1_Agree

Permitted2: [2-lane roadway] The bicyclist is permitted to ride in the center of the lane.
0_Disagree; 1_Agree

Safe2: [2-lane roadway] It is safe for the bicyclists to ride in the center of the lane.
0_Disagree; 1_Agree

BikeMoveRight4: [4-lane roadway] The bicyclist should move to the right and allow the following motorist to pass within the lane.
0_Disagree; 1_Agree

MVWait4: [4-lane roadway] The motorist behind the bicyclist should slow and wait for a break in incoming traffic before passing in the adjacent lane.
0_Disagree; 1_Agree

Permitted4: [4-lane roadway] The bicyclist is permitted to ride in the center of the lane.
0_Disagree; 1_Agree

Safe4: [4-lane roadway] It is safe for the bicyclists to ride in the center of the lane.
0_Disagree; 1_Agree

CycleDistance: How many miles do you bicycle during a typical week?
None; 1-10 miles; 11-50 miles; More than 50 miles

CycleDist1: CycleDistance recoded to numeric values.
0; 10; 50; 100

CycleDist2: CycleDistance recoded to less or more than 10 miles
<10 (None, 1-10 miles); >10 (11-50 miles, More than 50 miles)

MVDistance: How many miles do you drive a motor vehicle during a typical week?
None; Less than 50 miles; 51-200 miles; More than 200 miles

CommuteType: On a typical day, how do you get to and from work or school?
Bicycle; Personal motor vehicle (car, motorcycle, truck, etc.); Public transport (bus, school bus, train, etc.); Walk; More than one type of transport (for example, bicycle and bus); I do not commute to work or school; Other (specified)

CommuteType1: CommuteType recoded.
Most "Other responses" recoded to standard responses; several recoded to "Varies by day"

CommuteType2: CommuteType recoded to motor vehicle or something else.
Motor vehicle; Other

State: Standard two-character abbreviations for US state.

Gender: Sex of respondent.
Male; Female; no response (response not required to this question)

Education:
Did not graduate high school; High School; Community College; 4-Year College; Graduate Degree; no response (response not required to this question)
