# Supplementary material for: “Bicycles May Use Full Lane” Signage Communicates U.S. Roadway Rules and Increases Perception of Safety
Source: PLoS One. 2015 Aug 28;10(8):e0136973. doi: 10.1371/journal.pone.0136973 (PMC4552809; doi:10.1371/journal.pone.0136973)
Supplement: S1 Methods — Each screen from the survey is shown separately as the respondent would experience them. (PDF) [file pone.0136973.s003.pdf]

S1 Methods. Complete survey, shown here for "Bicycles May Use Full Lane" treatment. Each screen from the survey is shown separately, as the respondent would experience them.

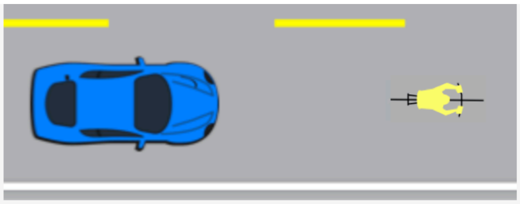

What Should They Do?

Informed Consent

This survey is part of a study designed to evaluate interactions among motorists and bicyclists on roadways in the United States. It should take less than 5 minutes to complete. Your participation is voluntary and you may stop at any time. There is no direct benefit to you for taking the survey, but your responses might help improve roadway design. Results will be posted at this site during October 2014.

We do not collect any identifying information, so your responses are anonymous. The protocol for data collection and storage has been reviewed and approved by NC State University's Institutional Review Board for the Protection of Human Subjects in Research (Study #4070).

By taking the survey you acknowledge that you have read this informed consent and are at least 18 years old.

We ask that you take the survey *only once* - but please retweet, send, or otherwise post the link to your friends - GO.NCSU.EDU/BIKECAR

Be sure to press *Submit* on the last page to record your responses.

If you have questions, comments, or concerns you may contact George Hess (details below).

Thank you for your time!

Proceed to the Survey

George R. Hess | Department of Forestry & Environmental Resources | NC State University | Raleigh NC 27695-8008 USA | 919.515.7437 | george_hess@ncsu.edu

Motorist - Bicyclist Interactions

* Required

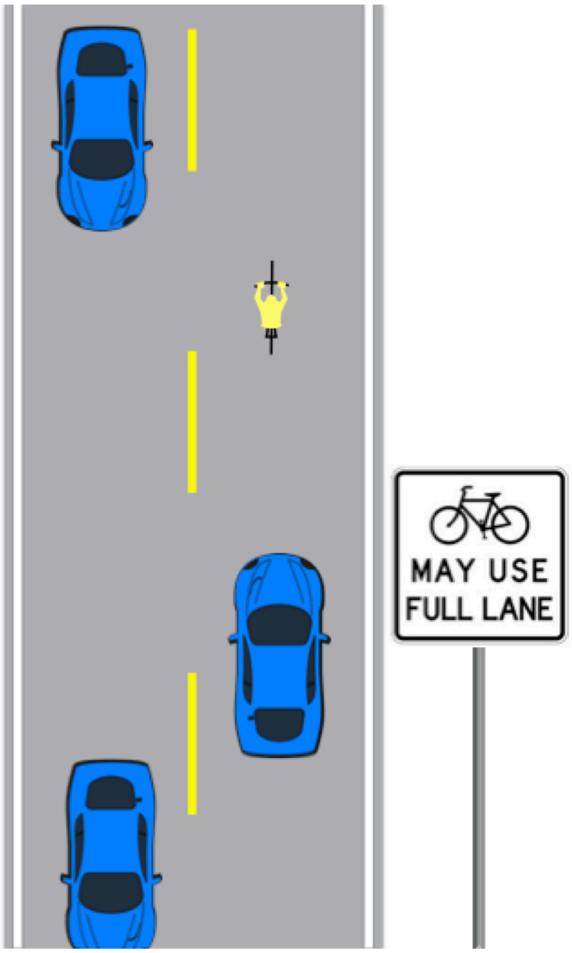

Please examine the situation above and respond to each of the four statements. *

	Agree	Disagree
The bicyclist should move to the right and allow the following motorist to pass within the lane.	○	○
The motorist behind the bicyclist should slow and wait for a break in oncoming traffic before passing in the adjacent lane.	○	○
The bicyclist is permitted to ride in the center of the lane.	○	○
It is safe for the bicyclist to ride in the center of the lane.	○	○

Continue »

25% completed

Motorist - Bicyclist Interactions

* Required

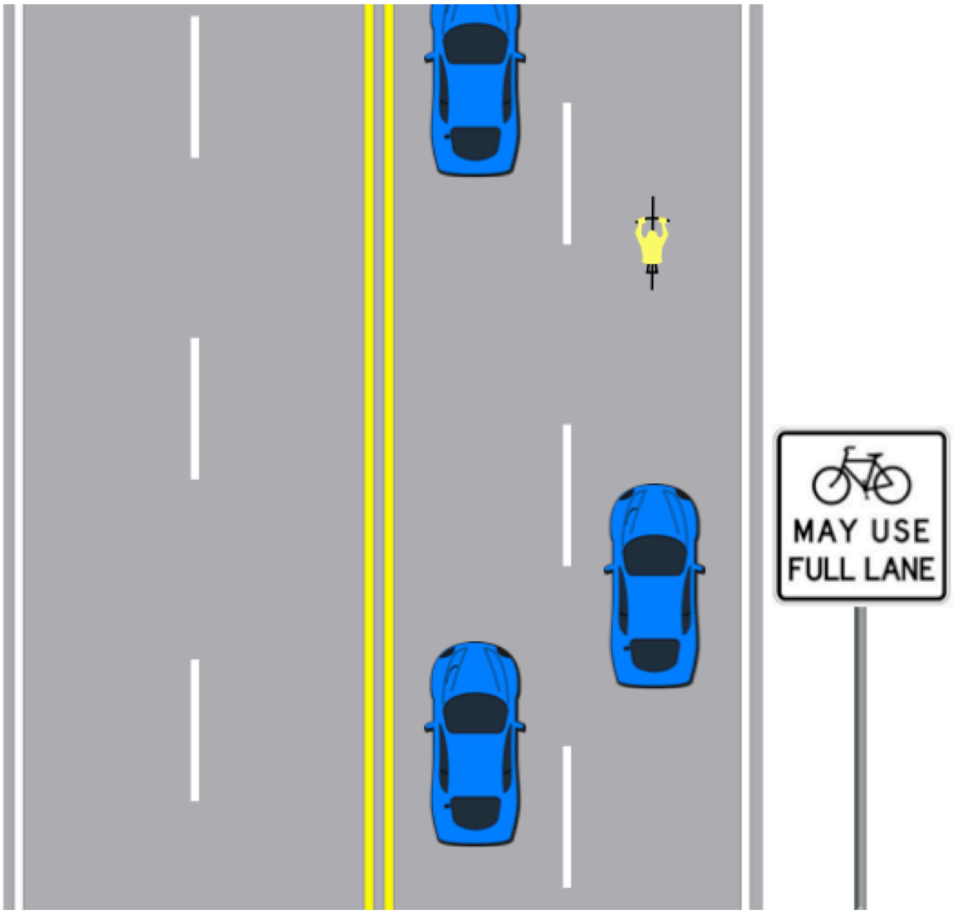

Please examine the situation above and respond to each of the four statements. *

	Agree	Disagree
The bicyclist should move to the right and allow the following motorist to pass within the lane.	○	○
The motorist behind the bicyclist should slow and wait for a break in traffic before passing in the adjacent lane.	○	○
The bicyclist is permitted to ride in the center of the lane.	○	○
It is safe for the bicyclist to ride in the center of the lane.	○	○

« Back   Continue »

50% completed

Motorist - Bicyclist Interactions

* Required

Transportation Information

Your answers to these 3 questions will help us interpret survey responses.

How many miles do you bicycle during a typical week? *

- ◯ None
- ◯ Less than 10 miles
- ◯ 11 - 50 miles
- ◯ More than 50 miles

How many miles do you drive a motor vehicle during a typical week? *

- ◯ None
- ◯ Less than 50 miles
- ◯ 51 - 200 Miles
- ◯ More than 200 Miles

On a typical day, how do you get to and from work or school? *

- ◯ Bicycle
- ◯ Personal motor vehicle (car, motorcycle, truck, etc.)
- ◯ Public transport (bus, school bus, train, etc.)
- ◯ Walk
- ◯ More than one type of transport (for example, bicycle and bus)
- ◯ I do not commute to work or school
- ◯ Other: ____________

« Back   Continue »

75% completed

Motorist - Bicyclist Interactions

* Required

Demographic Information

Your answers to these final 3 questions will help us interpret survey responses.
Please press "Submit" to record your responses.

What state do you live in? *

Please select your state from the list.

What is your gender?

- Male
- Female

What is your highest level of educational achievement?

- Did not graduate high school
- High School
- Community College
- 4-Year College
- Graduate Degree

Open Comment

Anything you'd like to say.

« Back | Submit

Never submit passwords through Google Forms.

100%: You made it.
