# Supplementary material for: “Bicycles May Use Full Lane” Signage Communicates U.S. Roadway Rules and Increases Perception of Safety
Source: PLoS One. 2015 Aug 28;10(8):e0136973. doi: 10.1371/journal.pone.0136973 (PMC4552809; doi:10.1371/journal.pone.0136973)
Supplement: S2 Methods — (PDF) [file pone.0136973.s004.pdf]

S2. Methods. Images for all four treatments, shown here for the two-lane roadway.

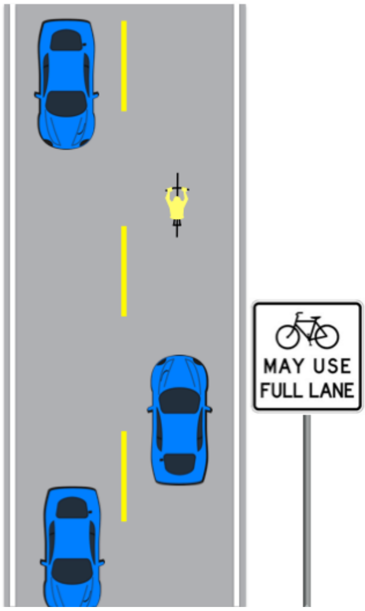

"Bicycles May Use Full Lane" signage.

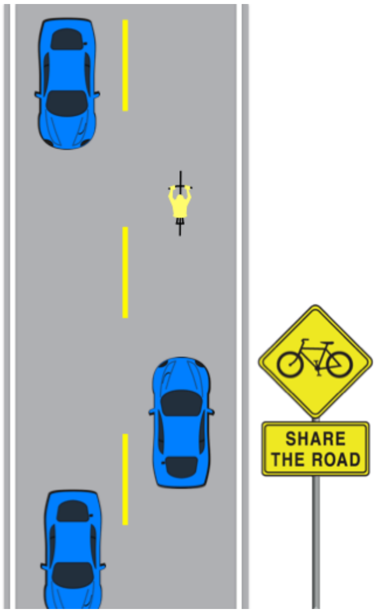

"Share the Road" signage.

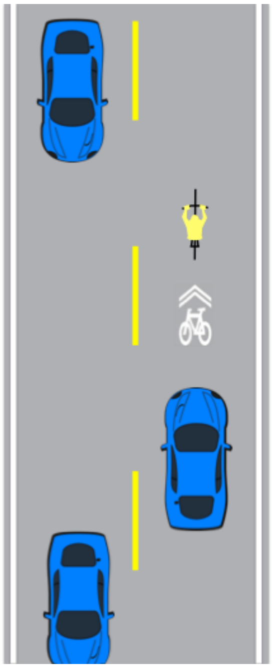

Shared Lane Markings on roadway pavement.

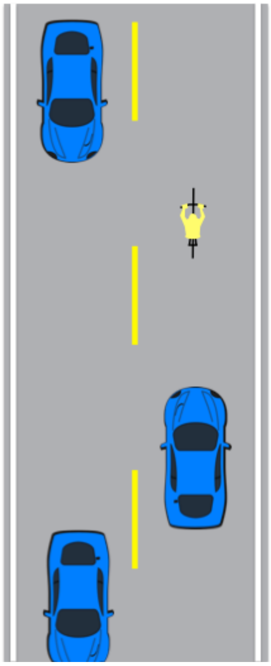

Control treatment – no bicycle-related signage or pavement markings.
