# Supplementary material for: “Bicycles May Use Full Lane” Signage Communicates U.S. Roadway Rules and Increases Perception of Safety
Source: PLoS One. 2015 Aug 28;10(8):e0136973. doi: 10.1371/journal.pone.0136973 (PMC4552809; doi:10.1371/journal.pone.0136973)
Supplement: S1 Table — (PDF) [file pone.0136973.s005.pdf]

# S1 Table. Parameter estimates, standard errors, and p-values for our logistic model.

Logistic model results shown for intercept, traffic device treatments (Share the Road, Shared Lane Markings, Bicycle May Use Full Lane), and user categories (respondents who cycle >16k/week vs. those who cycle≤16k/week; those who commute by means other than personal motor vehicle vs those who commute by personal motor vehicle). Each cell shows the parameter estimate, (standard error), and Pr(>|z|).

| | | *Treatments* | | | *User Categories* | |
|---|---|---|---|---|---|---|
| **Statement** | **Intercept** | **Share the Road** | **Shared Lane Markings** | **Bicycle May Use Full Lane** | **Cycle >16k/week** | **Commute by Other Than Motor Vehicle** |
| ***2-lane Road*** | | | | | | |
| The bicyclist should move to the right and allow to following motorist to pass within the lane. | -0.2092 (0.1362)<br>p=0.1247 | 0.1500 (0.1407)<br>p=0.2863 | -0.1189 (0.1412)<br>p=0.3997 | -0.3166 (0.1437)<br>p=0.0276 | -0.3688 (0.1272)<br>p=0.0037 | -0.3557 (0.1068)<br>p=0.0009 |
| The motorist behind the bicyclist should slow and wait for a break in oncoming traffic before passing in the adjacent lane. | 2.4021 (0.2870)<br>p<2E-16 | 0.1106 (0.3406)<br>p=0.7455 | 0.1253 (0.3352)<br>P=0.7086 | 0.8106 (0.4052)<br>p=0.0455 | 0.5718 (0.2872)<br>p=0.0465 | 0.5977 (0.2770)<br>p=0.309 |
| The bicyclist is permitted to ride in the center of the | 0.8915 (0.1705) | 0.1048 (0.1961) | 0.5276 (0.2101) | 0.6710 (0.2158) | 0.6648 (0.1679) | 0.7873 (0.1610) |

| | | | | | | |
|---|---|---|---|---|---|---|
| lane. | p=1.74E-07 | p=0.5930 | P=0.0120 | p=0.0019 | p=09.60E-05 | p=1.00E-06 |
| It is safe for the bicyclist to ride in the center of the lane | -0.2449 (0.1356) p=0.0710 | -0.1048 (0.1417) p=0.4594 | 0.1162 (1414) p=0.4113 | 0.2471 (0.1425) p=0.0828 | 0.7455 (0.1250) p=2.48E-09 | 0.5019 (0.1060) p=2.18E-06 |
| ***4-lane Road*** | | | | | | |
| The bicyclist should move to the right and allow to following motorist to pass within the lane. | -0.5824 (0.1460) p=6.67E-05 | -0.0100 (0.1514) p=0.9474 | -0.4685 (0.1590) p=0.0032 | -0.4723 (0.1584) p=0.0029 | -0.2455 (0.1394) p=0.0781 | -0.4200 (0.1186) p=0.0004 |
| The motorist behind the bicyclist should slow and wait for a break in traffic before passing in the adjacent lane. | 3.3946 (0.4394) p=1.11E-14 | 0.3785 (0.5221) p=0.4785 | 0.6476 (0.5534) p=0.2419 | 0.1809 (0.4800) p=0.7063 | 0.1152 (0.4397) p=0.7932 | 0.7865 (0.4032) 0.0511 |
| The bicyclist is permitted to ride in the center of the lane. | 1.1635 (0.1871) p=4.99E-10 | 0.0096 (0.2162) p=0.9645 | 0.6904 (0.2485) p=0.0055 | 0.6848 (0.2456) p=0.0053 | 0.7354 (0.1873) 8.64E-05 | 0.7863 (0.1845) 2.03E-05 |
| It is safe for the bicyclist to ride in the center of the lane. | -02397 (0.1371) p=0.0805 | 0.0034 (0.1438) p=0.9810 | 0.4676 (0.1482) p=0.0016 | 0.5178 (0.1484) p=0.0005 | 0.8458 (0.1275) p=3.25E-11 | 0.4270 (0.1109) p=0.0001 |
