# Supplementary material for: “Bicycles May Use Full Lane” Signage Communicates U.S. Roadway Rules and Increases Perception of Safety
Source: PLoS One. 2015 Aug 28;10(8):e0136973. doi: 10.1371/journal.pone.0136973 (PMC4552809; doi:10.1371/journal.pone.0136973)
Supplement: S1 Text — (PDF) [file pone.0136973.s006.pdf]

S1 Text. Review of studies of the three traffic control devices evaluated in this article.

In what follows, the word "significant" is used in the statistical sense, as reported by the authors.

Share the Road

In a 2014 review of bicycle related roadway measures, only a single evaluation of "Share the Road" signage was cited [1]. Kay and others performed a before-and-after observational study of motorist passing behavior on a two-lane rural highway in Michigan [2]. Signage was associated with a statistically significant increase in the number of motorists who moved left when passing bicyclists, but there was no significant increase in lateral distance between passing motor vehicles and bicycles or the number of passes with <1.52m (5ft) of lateral displacement. This study is not a true test of lane-sharing, however, because most (75%) of the bicyclists were riding in the 1.22m (4ft) paved shoulder adjacent to the 3.35m (11ft) travel lane, creating an effective operating space of 4.57m (15ft). In one additional study we found, Mondia and Duthi performed a before-and-after observation study on multi-lane urban roadways in Austin, TX, and found that "Share the Road" signage did not significantly influence bicyclist position within the travel lane, but did significantly increase the lateral distance between motorists

and bicyclists during passing [3]. Results may be confounded by the presence of sidewalks and pavement right of the travel lane, which many bicyclists used.

Bicycles May Use Full Lane

As part of a broader study, Brady and others observed motorists and bicyclist behavior before and after the installation of "Bicycles May Use Full Lane" signage on two multi-lane urban roadways in Austin, TX [4]. On one road, they observed a small (6.9cm (2.72in)) but statistically significant shift of average bicyclist position toward the center of the lane. The proportion of bicyclists riding in the center of the lane did not change significantly and the proportion of drivers who moved fully or partly into the adjacent lane increased significantly by more than 15 percentage points. On the other road, the proportion of bicyclists using the full lane significantly *decreased* by more than 20 percentage points *after* installation of the signage, and the proportion of cyclists riding on the sidewalk *increased* by more than 20 percentage points. They also revealed several situational and methodological issues that might confound their results, particularly extensive sidewalk use by bicyclists along one of their study routes. They noted that theirs was the first study of this signage, and we were unable to find any others.

Shared Lane Markings

Shared Lane Markings are the most studied of these three traffic control devices. There are at least a dozen evaluations, many reviewed by Mead and others [1]. The first was a

before-and-after study in Gainseville, Florida, that found a small (approximately 7.62cm (3in)), statistically significant increase in the lateral distance of bicyclists from the curb face and a smaller, statistically insignificant increase in lateral distance between bicycles and motor vehicles during passing [5]. Alta Planning+Design reported that installation of shared lane markings in San Francisco was followed by a statistically significant, 20.3cm (8in) increase in distance between bicyclists and parked vehicles and a significant increase of 0.61-0.69m (2-2.25ft) between passing motorists and bicyclists [6]. Mondia & Duthie found that shared lane markings increased the distance bicyclists drove from the curb and led to more lateral space between motorists passing bicyclists [3].

Hunter and others conducted before-and-after evaluations in three cities, with mixed results [7]. On a 4-lane roadway with a concrete median in Cambridge, MA, Shared Lane Markings were centered 3.05m (10ft) from the curb in a 6.40m (21ft) wide outer lane that included on-street parking. After the Shared Lane Markings were installed, significantly *fewer* bicyclists took control of the lane (used the full lane) to prevent motorists from passing and significantly *fewer* motorists moved fully or partially into the adjacent lane to pass. Later spacing between motorists passing bicyclists did not change significantly. On a 5-lane roadway (center turning lane) in Chapel Hill, NC, Shared Lane Markings were installed (3.63ft) from the curb of a 4.57m (15ft) (including the 0.61m (2ft) gutter pan) outer lane. Again, significantly *fewer* motorists moved fully or partially into the adjacent lane to pass. Very few bicyclists took control of the lane before (2%) or after (1%) installation, and the difference was not statistically significant. Lateral spacing between motorists passing bicyclists *decreased* significantly in the downhill direction.

On a two-lane roadway in Seattle, WA, with parking on both sides of the street, the downhill lane was restriped from 20ft to 17.5ft with a Shared Lane Marking centered in the travel lane, 12.25ft from the curb (a bicycle lane was striped on the uphill side of the roadway). The lane-changing behavior of motorists did not change significantly after installation, nor did the proportion of bicyclists who drove in the center of the travel lane (27% before, 25% after). Interestingly, the proportion of bicyclists driving in the center of the lane increased from to 51% after the lane had been narrowed but before the Shared Lane Markings were installed. Lateral spacing between motorists passing bicyclists was not reported.

It is difficult to draw general conclusions from these findings with respect to bicyclists using the full lane, in part because Shared Lane Markings rarely were placed in the center of the travel lane. Instead, they were most often positioned to the right of center, including directly adjacent to the curb. Pein (2010, 2011, 2012b) has criticized many of these studies, including repudiating his own 1999 study [5], because the Shared Lane Markings evaluated were placed improperly [8-10]. He noted that placement is often in contradiction to recommendations in the Manual on Uniform Traffic Control Devices [11] and that Shared Lane Markings are being used as “pseudo-bicycle lanes” within travel lanes too narrow to be restriped with proper bicycle lanes. As such, they encourage bicyclists to ride within door zones, and side-by-side lane sharing by motorists and bicyclists in lanes too narrow to accommodate such interactions [10]. In response to this concern, the recently revised Institute of Transportation Engineers’ Traffic Control Devices Handbook suggests placement of Shared Lane Markings near the center of the

travel lane in situations where space is inadequate for same-lane passing [12]; we followed this guidance in our study.

References

1. Mead J, McGrane A, Zegerr C, Thomas L (2014) Evaluation of Bicycle-Related Roadway Measures: A Summary of Available Research. Chapel Hill, NC: Pedestrian and Bicycle Information Center. Available: http://katana.hsrc.unc.edu/cms/downloads/06%2013%202014%20BIKESAFE%20Lit%20Review_FINAL.pdf. Accessed 10 December 2014.

2. Kay J, Savolainen PT, Gates TJ (2013) An Evaluation of the Impacts of a Share the Road Sign on Driver Behavior Near Bicyclists. http://assets.conferencespot.org/fileserver/file/41200/filename/2vc827.pdf. Accessed 5 December 2014.

3. Mondia JJ, Duthi JC (2012) Analysis of factors influencing bicycle-vehicle interactions on urban roadways by ordered probit regression. Transportation Research Record" Journal for the Transportation Research Board 2314: 81-88.

4. Brady JF, Loskorn J, Mills AF, Duthie J, Machemehl RB (2011) Operational and safety implications of three experimental cicyle safety devices in Austin, TX. 90th Annual Meeting of the Transportation Research Board. Available: http://docs.trb.org/prp/11-0921.pdf. Accessed 17 December 2014.

5. Pein W, Hunter WW, Stewart JR (1999) Evaluation of the Shared-Use Arrow. Report to Florida Department of Transportation. Available: http://nacto.org/wp-content/uploads/2011/01/Evaluation-of-the-Shared-Use-Arrow.pdf. Accessed 17 December 2014.

6. Alta Planning+Design (2004) San Francisco's Shared Lane Pavement Markings: Improving Bicycle Safety, Final Report. Available: http://nacto.org/wp-content/uploads/2010/08/San-Franciscos-Shared-Lane-Pavement-Markings-Improving-Bicycle-Safety.pdf. Accessed 30 December 2014.

7. Hunter WW, Thomas L, Srinivasan R, Martell C (2010) Evaluation of Shared Lane Markings. Publication FHWA-HRT-10-041. FHWA, U.S. Department of Transportation. Available: http://www.fhwa.dot.gov/publications/research/safety/pedbike/10041/10041.pdf. Accessed 30 December 2014.

8. Pein W (2010) Critique of: "San Francisco's Shared Lane Pavement Markings: Improving Bicycle Safety."

https://bicyclingmatters.files.wordpress.com/2010/01/critique-of-san-fran-shared-use-report19.pdf. Accessed 17 December 2014.

9. Pein W (2011) Critique of: "Evaluation of the Shared-Use Arrow." http://bicyclingmatters.files.wordpress.com/2011/04/critique-of-my-slm-paper1.pdf. Accessed 17 December 2014.

10. Pein W (2012) Critique of: Evaluation of Shared Lane Markings. https://bicyclingmatters.files.wordpress.com/2011/06/critique-of-eval-of-slms28.pdf. Accessed 17 December 2014.

11. US Departmant of Transportation-Federal Highway Administration (2012) Manual on Uniform Traffic Control Devices for Streets and Highways, 2009 Edition, Including Revisions 1 & 2 dated May 2012. http://mutcd.fhwa.dot.gov/pdfs/2009r1r2/mutcd2009r1r2edition.pdf. Accessed 10 December 2014.

12. Seyfried RK, editor (2013) Traffic Control Devices Handbook, 2nd Edition. Washington, DC: Institute of Transportation Engineers.
